# Supplementary material for: Pneumatosis Intestinalis Induced by Alpha-Glucosidase Inhibitors in Patients with Diabetes Mellitus
Source: J Clin Med. 2022 Oct 7;11(19):5918. doi: 10.3390/jcm11195918 (PMC9571713; doi:10.3390/jcm11195918)
Supplement: Supplementary file 1 [file jcm-11-05918-s001.zip › jcm-1932384-supplementary.pdf]

Supplemental Table S1. Clinical features of patients without comorbidities (N = 10).

Reference	Age /Sex	Diabetes mellitus duration	Concomitant medications	Alpha-glucosidase/ dose/ duration	Symptoms	Locations	Gas in cavities	Surgery	Recovered (radiology confirmed)
Hayakawa, et al., 1999 [13]	64/F	DM II/20 years	Insulin; antilipemic agents	Voglibose/ 0.6 mg daily / 2 months	Abdominal distension	Ascending and proximal transverse colon.	x	x	4 days
Yanaru, et al., 2002 [14]	61/M	Unknown	Sulfonylurea	Voglibose/ 0.6mg daily/5 years	Abdominal distension, constipation, hematochezia	Sigmoid colon	x	x	28 days
Furio, et al., 2006 [15]	64/F	Unknown	Insulin	Acarbose/ unknown/ 3 years	Abdominal pain, diarrhea, tenesmus, weight loss	Cecum, ascending colon, transverse colon, sigmoid colon	x	x	15 days
Kojima, et al., 2010 [12]	58/M	Unknown	N/A	Miglitol/ 150 mg daily/ 8 months	Abdominal pain, rectal bleeding	Ileocecal region, ascending colon	Pneumoperitoneum, Pneumoretroperitoneum	x	12 days
Wu & Bao, 2011 [16]	67/F	Unknown	N/A	Acarbose/ unknown/ unknown	N/A	Ascending colon	x	x	3 months
Tseng, et al., 2011 [17]	76/F	Unknown	N/A	α-glucosidase inhibitor (undefined)/ unknown/ 5 years	Abdominal pain, chest pain, dyspnea	Ascending colon	x	Laparotomy and hemicolectomy	Unknown
Takase, et al., 2017 [18]	78/F	3 years	Sitagliptin	Voglibose/ 0.6 mg daily/ 2 weeks	Abdominal pain	Small intestine	x	x	11 days
Liao, et al., 2017 [19]	50/M	Unknown	N/A	Acarbose/ 150 mg daily/ 1 year	Abdominal pain, fever, diarrhea	All colon	x	x	Undefined
Wang, et al., 2018 [20]	72/F	10 years	Insulin	Acarbose/ unknown/ 10 years	Constipation then diarrhea, abdominal distension, bloody and mucous stool	Ascending colon; sigmoid colon	x	x	6 months
Lin and Wang, 2019 [21]	65/M	Unknown	N/A	Acarbose/unknown/ 3 years	Positive fecal occult blood test	Sigmoid colon	x	x	6 months

Supplemental Table S2. Clinical features of patients with comorbidities (N = 19)

Reference	Age/Sex	Diabetes' duration	Diseases other than diabetes	Concomitant medications	Alpha-glucosidase/ dose/duration	PI symptoms	Locations	Gas in cavities 11/17	Surgery	PI disappeared
Azami, 2000 [22]	87/F	14 years	Hypothyroidism; paralytic ileus	Glibenclamide; maltitol	Acarbose/150mg daily/ 2 years	Abdominal distension, low appetite	Small intestine	x	x	5 days
Hisamoto, et al., 2006 [23]	56/F	7 days	Non-specific interstitial pneumonitis (NSIP)	Prednisone	Voglibose/600mg daily/7 days	N/A	Terminal ileum, ascending colon, transverse colon	Pneumomediastinum, Pneumopericardium, Pneumoretroperitoneum	x	7 days
Maeda, et al., 2006 [24]	72/ F	Unknown	Minimal change nephrotic syndrome; *E. Coli* sepsis	Prednisolone; mizoribine; insulin	Voglibose/ 0.9 mg daily/3 years	Abdominal pain	Small intestine (DIC, acute renal and pulmonary failure)	x	x	Undefined
Saito, et al., 2007 [25]	53/F	1 month	Dermatomyositis	Prednisolone; methotrexate; insulin	Voglibose /0.6 mg/ 1 month	Abdominal flatulence, abdominal pain, nausea, a sense of irritation on the right neck	Ascending, descending colons, mesentery	Subcutaneous air in the cervical region, pneumomediastinum, pneumoperitoneum, pneumoretroperitoneum	x	21 days
Tsujimoto, et al., 2008 [26]	69/M	2.5 years	Myasthenia gravis	Prednisolone; sulfonylurea	Voglibose /unknown/ 1 year and 8 months	Abdominal distension, constipation, bright rectal bleeding	Sigmoid colon	Pneumoperitoneum	x	2 weeks
Vogel, et al., 2009 [27]	65/F	12 years	Hypertension; post-hysterectomy/ovariectomy	N/A	Acarbose/ 150 mg daily/12 years	Abdominal pain	Ascending colon	Pneumoperitoneum	x	3 months
Shimojima, et al., 2011 [28]	48/M	4 weeks	Neuropsychiatric systemic lupus erythematosus (NPSLE)	Prednisolone; glimepiride	Voglibose X 2 weeks	N/A	Ascending colon	x	x	13 weeks
Imai, et al., 2012 [29]	87/M	Unknown	Hypertension	N/A	Miglitol/225mg daily/7 years	Abdominal pain, distension, nausea	Small intestine	Pneumoperitoneum, ascites	x	7 days
Tanabe, et al., 2013 [30]	80/F	Unknown	Hypertension; post cerebral infarction with right-side paralysis; post-cholecystectomy and hysterectomy	Antihypertensive (undefined)	α-glucosidase inhibitor (undefined)	Abdominal bloating, abdominal pain	Small intestine	Pneumoperitoneum	x	Unknown
Makiyama, et al., 2014 [31]	80/F	2 days	Acute cholecystitis	N/A	Voglibose/ 0.6 mg daily/ x 2 days	Abdominal distention, vomiting	Terminal ileum	Portal venous gas	Exploratory laparotomy	Undefined
Ogo, et al., 2014 [32]	83/F	Unknown	Polymyalgia rheumatica	Prednisone; insulin	Acarbose/100mg t.i.d./unknown	N/A	Ascending colon, transverse colon	pneumoperitoneum,	x	3 weeks
Rottenstreich, et al., 2015 [33]	73/M	Unknown	Hypertension; ischemic heart disease; diabetic nephropathy	N/A	Acarbose/50 mg t.i.d./unknown	Vomiting, constipation	Ileum, ascending colon	Portal venous gas, pneumoperitoneum	Exploratory laparotomy	Undefined
Ksiadzyna & Peña 2016 [4]	64/M/	8 Years	Chronic mild non-specific colitis	Metformin	Acarbose/150 mg daily/8 years	Watery diarrhea, flatulence, abdominal pain	Cecum, splenic flexure	x	x	4 weeks
Suzuki, et al., 2017 [34]	70/F	10 years	Rheumatoid arthritis	Prednisolone	Voglibose/ undefined/10 years	N/A	Colon	Pneumoperitoneum	x	2 weeks
Suzuki, et al., 2017 [34]	71/F	<1 year	Granulomatosis with polyangiitis	Prednisolone	Voglibose/unknown/1 year	Nose pain	Intestinal wall (undefined)	Pneumoperitoneum	x	1 month
Ling, et al., 2019 [35]	64/M	5 years	Chronic inflammatory colitis	N/A	Acarbose/unknown/5 years	Bloody stool	Sigmoid	x	x	3 months
Police, et al., 2020 [36]	72/undefined	10 years	Sigmoid volvulus; dolichocolon; atrial fibrillation	N/A	Acarbose/unknown/10 years	Abdominal pain, acute occlude syndrome	Sigmoid	x	Laparoscopic sigmoidectomy	Undefined

Oda, et al., 2021 [37]	70/F	Unknown	Hypertension; cardiovascular disease, post cerebral infarction; chronic kidney failure due to diabetic nephropathy; peritoneal dialysis-peritonitis; nonocclusive mesenteric ischemia (NOMI)	Linagliptin; clopidogrel; aspirin; amlodipine; olmesartan; carvedilol	Voglibose/0.9 mg daily/unknown	Diarrhea, chill, low appetite, abdominal pain, Bp 86/50	Ascending colon (with hemorrhage and ulcers in the entire right colon and the terminal ileum)	Portal venous gas	x	4 days
Otsuka, et al., 2021 [38]	59/M	4 years	Post lung transplantation for lung cancer and idiopathic pulmonary fibrosis 1031 days; pneumonia 47 days ago	Tacrolimus, prednisolone, insulin, oral acyclovir	Voglibose/0.9 mg daily/4 years	N/A	Ascending colon, transverse colon, descending colon	Pneumoperitoneum Pneumoretroperitoneum	x	11 days

Abbreviation: t.i.d. = three time a day; DIC = Disseminated Intravascular Coagulation.
